# Incidence, risk factors and treatment of diarrhoea among Dutch travellers: reasons not to routinely prescribe antibiotics

**DOI:** 10.1186/1471-2334-11-295

**Published:** 2011-10-29

**Authors:** Sanne-Meike Belderok, Anneke van den Hoek, Joan A Kint, Maarten F Schim van der Loeff, Gerard JB Sonder

**Affiliations:** 1 Department of Infectious Diseases, Public Health Service (GGD), Amsterdam, The Netherlands; 2 Department of Internal Medicine, Division of Infectious Diseases, Tropical Medicine and AIDS, Academic Medical Centre, Amsterdam, The Netherlands; 3 National Coordination Centre for Traveler's Health Advice (LCR), Amsterdam, The Netherlands

## Abstract

**Background:**

Travellers' diarrhoea (TD) is the most common infectious disease among travellers. In the Netherlands, stand-by or prophylactic antibiotics are not routinely prescribed to travellers. This study prospectively assessed the incidence rate, risk factors, and treatment of TD among immunocompetent travellers.

**Methods:**

Persons who attended the travel clinic of the Public Health Service Amsterdam in 2006-2007 before short-term travel to tropical and subtropical countries were invited to answer a questionnaire regarding sociodemographics and travel purpose; they were also asked to keep a daily structured travel diary, recording their itinerary, symptoms, and self-medication or consultation with a doctor. Diarrhoea episodes containing blood or mucous were considered severe.

**Results:**

Of 1202 travellers, the median age was 38 years, and the median travel duration 3 weeks. Of all episodes, 96% were mild. The median duration of TD was 2 days and significantly shorter in subsequent episodes compared to first episodes (p < 0.0005). Of first episodes 38% started in the first travel week. The incidence rate (IR) for first episodes was 2.49 (95% confidence interval [CI], 2.30-2.70) per 100 travel days, with the highest IR among travellers to South-Central and West Asia. The IR for first and subsequent episodes was comparable. Risk factors for first episodes included female sex, a Western country of birth, and tourism as the purpose of travel. The lowest risk was in travellers to South America. An independent risk factor for subsequent episodes was female sex. In total, 5% of travellers used antibiotics; of those, 92% had mild diarrhoea, and 53% received antibiotics over the counter.

**Conclusions:**

TD is common among travellers, but the overall course is mild, not requiring treatment. The incidence rates for first and second episodes are comparable. Female sex is a risk factor for the first episode, as well as subsequent ones. Prescription antibiotics are not needed in short-term healthy travellers.

## Background

International tourism in 2007 showed approximately 908 million tourist arrivals worldwide [1]. Among tourists from industrialized countries, about 80 million crossed the borders of tropical and subtropical countries, many of which are developing areas, each year [2]. The most common infectious health problem among travellers from industrialized regions to developing countries is travellers' diarrhoea (TD) [2-11]. Because of the continuing high rates of international travel, TD will continue to be an important problem.

The annual number of Dutch travellers to tropical and subtropical countries doubled from approximately 1 million in 1999 to about 2 million in 2007 [12]. The Netherlands has a restrictive antibiotic policy and, therefore, low microbial resistance patterns [13-15]. The Dutch national guidelines for traveller's health advice do not advise everyone to carry stand-by treatment, only those at increased risk, such as immunocompromised persons and travellers to very remote areas [16].

In this prospective study we estimated the attack rate and incidence rate and studied risk factors and characteristics of TD among a cohort of immunocompetent, short-term travellers to tropical and subtropical countries. Further, we described treatment and the severity of episodes.

## Methods

### Study population

**Figure 1:**
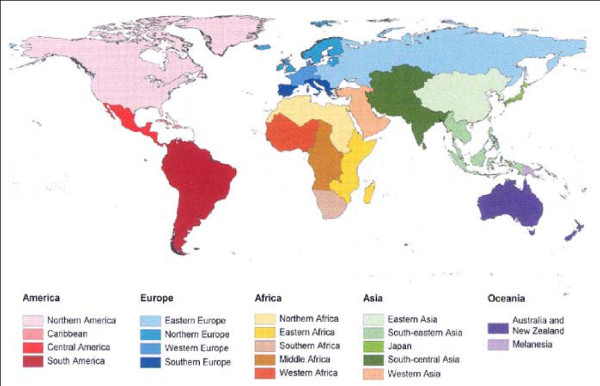
**UN classification of the world, in regions (2002)**.

A prospective study recruited persons attending the travel clinic of the Public Health Service Amsterdam from October 2006 to October 2007 [17]. All immunocompetent persons 18 years and older were eligible if they were planning to travel for 1 to 13 weeks to one or more developing countries. Based on the definition by the United Nations, Department of Economics and social affairs [18], we categorized these countries in 6 regions: South America; Central America and Caribbean; Middle, Western and Northern Africa; Southern and Eastern Africa; South-Eastern and Eastern Asia; and South-Central and Western Asia (Figure 1). Those who reported a history of immunosuppressive disorder, with functional gastrointestinal disorders, and pregnant women were excluded.

All participants were seen by a medical doctor or nurse specialised in travel medicine. Based on Dutch national guidelines on traveller's health advice, they received vaccinations, a prescription for antimalarial chemoprophylaxis if required, and oral and written information about how to avoid acquiring travel-related diseases including diarrhoea, such as good personal hygiene and sanitary conditions and avoiding unsafe water and food. For diarrhoea, travellers were advised to carry an antimotility agent and oral rehydration solution. Cholera vaccine is not indicated, according to the guidelines. Antibiotics, which cannot be purchased without a prescription in the Netherlands, are only prescribed for the treatment of TD to those at increased risk, and thus, a prescription for these immunocompetent travellers was not provided.

### Survey methods

A standard questionnaire in Dutch or English was used to collect data before departure on sociodemographics, travel history and purpose of the travel (tourism, work or education, or visiting friends and/or relatives). Participants were given a thermometer (ordered 2006, Cemex, Bleiswijk, The Netherlands) and asked to take their temperature when feeling feverish. They were also asked to keep a structured travel diary, recording symptoms, itinerary, and signs of disease, such as a gastrointestinal disorder (diarrhoea, fever, vomiting, bloody or mucous stools or other symptoms) and possible self-treatment or involvement of a doctor during a diarrhoeal episode. Participants made daily diary entries from the day they arrived at their destination to 1 week after their return, to encompass incubation periods of diarrhoea. Thus, 'travel-related' in our study refers to the period of travel and one week thereafter. Travel duration was recorded as the total days spent in areas meeting the inclusion criteria. All participants were seen between 2 and 6 weeks after return, when the diary was checked by a registered nurse in the participant's presence. Participants received a gift voucher of 25€ for completing the study. The study protocol was approved by the Medical Ethics Committee of the Academic Medical Center Amsterdam. Participants were included only with informed and written consent.

### Definitions

Diarrhoeal episodes were regarded as TD when they met the definition of the World Health Organization [19] (any episode of any number of more frequent passage of loose or liquid stools per day than is normal for the individual from the beginning of the journey to the end of the first week after return). An episode was regarded as severe when the diarrhoea contained blood, mucous, or both. The episodes were considered separate if they were at least 5 days apart [3]. Age was categorized in 3 groups, because age 18-30 years was shown to be a risk factor for TD [6,11]. Travellers older then 30 years were divided into two other age groups (31 - 45 and 46 - 80 years).

### Data analysis

Data analysis was performed with SPSS version 17.0.2 (2009, IBM, Somers, USA) and version 19.0.0.1 (2010), Stata statistical software, version 11 (Statacorp, College Station, USA) and OpenEpi (version 2.3, May 2009, Andrew G. Dean and Kevin M. Sullivan, Atlanta, USA).

Incidences were expressed both as attack rates (ARs) and as incidence rates (IRs). Attack rates were calculated by dividing the number of persons reporting episodes of TD by the total number of persons at risk. Incidence rates per 100 travel days for the first episode were calculated by dividing the number of first episodes by the total number of travel days in which participants were at risk for a first episode. Incidence rates for subsequent episodes were calculated by dividing the number of second or later episodes by travel days at risk. Only travellers who had experienced a first episode contributed to this analysis, and time at risk started 5 days after the last day of the first episode. Travel destination was treated as a variable with six categories, the reference category being the geographical region with the lowest attack rate for TD. Independent risk factors were identified by multiple logistic regression analysis and univariate and multivariate Poisson analysis. Both in the univariate analysis and the multivariate analysis, associations were expressed as incidence rate ratios. Age and sex were forced into the Poisson model. A p-value < 0.05 was considered statistically significant.

## Results

### Study population

Between October 2006 and October 2007 1276 subjects who intended to travel to the developing world provided informed consent. Of these, 74 (6%) were excluded after the study ended: 23 had their travel arrangements cancelled and 42 were lost to follow-up. For this study we excluded 9 other subjects: 5 with functional gastro-intestinal disorders, 3 pregnant women, and 1 with an immune disorder. The remaining 1202 formed the study population (characteristics are shown in Table 1). The median age was 38 years (40 for men, 37 for women). The vast majority were native Dutch tourists travelling on holiday; 8% travelled for work or education, and 6% were visiting friends or relatives. The median travel duration was 3 weeks. The most frequently visited continent was Asia (47%); 28% travelled to Latin America and 25% to Africa. Most of the 1202 travellers had visited subtropical and tropical countries before (979, 81%).

**Table 1:** Characteristics of a prospective cohort of 1202 travellers to developing countries who attended a Dutch travel health clinic for pre-travel advice, October 2006 - October 2007

Number of subjects	1202	
Male sex	520	43%
Median age in years°	38	29-51
Age groups in years		
18 - 30	394	33%
31 - 45	379	32%
46 - 80	429	36%
Country of birth		
Netherlands	1048	87%
Other Western country	67	6%
Non-Western country	87	7%
Previous travel to a developing country		
Never	223	19%
1 - 5 times	698	58%
6 times or more	281	23%
Previous travel destinations*^1^		
Asia	664	55%
Latin America	543	45%
Africa	524	44%
Primary purpose of travel		
Tourism	1030	86%
Work or education	98	8%
Visiting friends and/or relatives	74	6%
Current travel destination (regions)		
South-Eastern and Eastern Asia	422	35%
South-Central and Western Asia	127	11%
South America	202	17%
Central America and Caribbean	120	10%
		
Southern and Eastern Africa	184	15%
Eastern Africa	119	10%
Southern Africa	45	4%
Both Eastern and Southern Africa	20	2%
Middle, Western and Northern Africa	111	9%
		
Several regions*^2^	33	3%
Several continents*^2^	3	
		
Median travel duration in days°	21	15-28
< 21 days	527	44%
21-34 days	533	44%
≥ 35 days	142	12%

° Interquartile range in second column

*^1 ^Does not add up to 100%, because persons travelled to more than one continent

*^2 ^Visited more than 1 region or continent during travel

### Attack rates and incidence rates

Of all 1202 travellers, 597 (AR 50%, 95% CI 47-52) experienced one or more episodes of diarrhoea (Table 2). In total, 781 episodes were reported by 597 travellers. Most of the 1202 travellers (450, 37%) reported only 1 episode; 121 (10%) reported 2 episodes, 19 (2%) 3 episodes, 3 (0.2%) 4 episodes, and 4 (0.3%) reported 5 episodes. Both the attack rate and the incidence rate for the first episode were highest in travellers to South-Central and Western Asia (the Indian subcontinent) (AR: 62%; IR: 3.83 per 100 travel days [95% CI 3.07 - 4.77]). Independent risk factors for a first episode of TD were: female sex, a Western country of birth, travel as a tourist as compared to visiting friends and relatives, and travel to South-Central and Western Asia (the Indian subcontinent), Central America and Caribbean, and Middle, Western and Northern Africa as compared to South America.

**Table 2:** Attack rates, incidence rates and predictors of first episodes of TD in a prospective cohort of 1202 travellers from the Netherlands to developing countries, October 2006 - October 2007

			≥ 1TD episode	Attack rate	Person-days of travel	Crude Incidence Rate per 100 travel days (95%CI)	Incidence Rate Ratio, univariate (95%CI)	P value	Incidence Rate Ratio, multivariate (95%CI)	P value
Total		1202	597	50%	23 959	2.49 (2.30 - 2.70)				

Sex										
	Male	520	236	45%	10714	2.20 (1.94 - 2.50)	1.00	**0.011**	1.00	**0.013**
	Female	682	361	53%	13245	2.73 (2.46 - 3.02)	1.24 (1.05 - 1.46)		1.23 (1.04 - 1.46)	
										
Age group, years										
	18-30	394	220	56%	7663	2.87 (2.52 - 3.28)	1.00	**0.023**	1.00	0.058
	31-45	379	171	45%	7846	2.18 (1.88 - 2.53)	0.76 (0.62 - 0.93)		0.78 (0.64 - 0.96)	
	46-80	429	206	48%	8450	2.44 (2.13 - 2.80)	0.85 (0.70 - 1.03)		0.87 (0.72 - 1.06)	
										
Country of birth										
	Netherlands	1048	535	51%	20517	2.61 (2.40 - 2.84)	1.00	**0.003**	1.00	**0.009**
	Other Western country	67	34	51%	1487	2.29 (1.63 - 3.20)	0.88 (0.62 - 1.24)		0.83 (0.59 - 1.18)	
	Non-Western country	87	28	32%	1955	1.43 (0.99 - 2.08)	0.55 (0.38 - 0.80)		0.58 (0.39 - 0.86)	
										
Primary purpose of travel										
	Tourism	1030	518	50%	20306	2.55 (2.34 - 2.78)	1.00	**0.005**	1.00	**0.032**
	Work or education	98	53	54%	1854	2.86 (2.18 - 3.74)	1.12 (0.85 - 1.49)		1.12 (0.84 - 1.51)	
	Visiting friends and/or relatives	74	26	35%	1799	1.45 (0.98 - 2.12)	0.57 (0.38 - 0.84)		0.63 (0.42 - 0.94)	
										
Previous travel to a developing country										
	Never	223	118	53%	4178	2.82 (2.36 - 3.38)	1.00	0.109		
	1 to 5 times	698	351	50%	13881	2.53 (2.28 - 2.81)	0.90 (0.73 - 1.10)			
	6 times or more	281	128	46%	5900	2.17 (1.82 - 2.58)	0.77 (0.60 - 0.99)			
										
Travel destination										
Latin America	South America	202	88	44%	4483	1.96 (1.59 - 2.42)	1.00	**< 0.0005**	1.00	**< 0.0005**
	Central America and Caribbean	120	66	55%	2014	3.28 (2.58 - 4.17)	1.67 (1.21 - 2.30)		1.54 (1.12 - 2.13)	
Africa	Southern and Eastern Africa	184	85	46%	3804	2.24 (1.81 - 2.76)	1.14 (0.85 - 1.53)		1.10 (0.82 - 1.49)	
	Middle, Western and Northern Africa	111	62	56%	1747	3.55 (2.77 - 4.55)	1.81 (1.31 - 2.50)		1.93 (1.39 - 2.68)	
Asia	South-Eastern and Eastern Asia	422	199	47%	8811	2.26 (1.97 - 2.60)	1.15 (0.90 - 1.48)		1.11 (0.87 - 1.43)	
	South-Central and Western Asia	127	79	62%	2063	3.83 (3.07 - 4.77)	1.95 (1.44 - 2.64)		1.94 (1.43 - 2.64)	
Several regions	Several regions	33	15	46%	975	1.54 (0.93 - 2.55)	0.78 (0.45 - 1.36)		0.75 (0.44 - 1.30)	

The median duration of both the first and subsequent episode was 2 days (IQR_first episode _1-6, IQR_subsequent episode _1-3; p < 0.0005).

The incidence rates for first and subsequent episodes were comparable; 2.49 (95% CI 2.30 - 2.70) for first episodes (Table 2) and 2.75 (95% CI 2.38 - 3.18) for subsequent episodes (Table 3).

**Table 3:** Incidence rates of and predictors for acquiring 2^nd ^or later TD episodes among 597 travellers in a prospective cohort from the Netherlands to developing countries, October 2006 - October 2007

Diarrhoea		Subsequent episodes	Person-days of travel	Crude Incidence Rate per 100 travel days (95%CI)	Incidence Rate Ratio, univariate (95%CI)	P value	Incidence Rate Ratio, multivariate (95%CI)	P value
Total		184	6690	2.75 (2.38 - 3.18)				

Sex								
	Male	57	2687	2.12 (1.64 - 2.75)	1.00	**0.010**	1.00	**0.009**
	Female	127	4003	3.17 (2.67 - 3.78)	1.50 (1.09 - 2.04)		1.50 (1.10 - 2.05)	
								
Age group, years								
	18-30	80	2945	2.72 (2.18 - 3.38)	1.00	0.534	1.00	0.504
	31-45	45	1824	2.47 (1.84 - 3.30)	0.91 (0.63 - 1.31)		0.93 (0.65 - 1.34)	
	46-80	59	1921	3.07 (2,38 - 3,96)	1.13 (0.81 - 1.58)		1.16 (0.83 - 1.63)	
								
Country of birth								
	Netherlands	168	6091	2.76 (2.37 - 3.21)	1.00	0.555		
	Other Western country	10	294	3.40 (1.83 - 6.32)	1.23 (0.65 - 2.33)			
	Non-Western country	6	305	1.97 (0.88 - 4.38)	0.71 (0.32 - 1.61)			
								
Primary purpose of travel								
	Tourism	156	5649	2.76 (2.36 - 3.23)	1.00	0.658		
	Work or education	24	823	2.92 (1.96 - 4.35)	1.06 (0.69 - 1.62)			
	Visiting friends and/or relatives	4	218	1.84 (0.69 - 4.89)	0.66 (0.25 - 1.79)			
								
Previous travel to a developing country								
	Never	25	1344	1.86 (1.26 - 2.75)	1.00	0.069		
	1 to 5 times	122	4082	2.99 (2.50 - 3.57)	1.61 (1.05 - 2.47)			
	6 times or more	37	1264	2.93 (2.12 - 4.04)	1.57 (0.95 - 2.61)			
								
Travel destination								
Latin America	South America	27	875	3.09 (2.12 - 4.50)	1.00	0.064		
	Central America and Caribbean	8	472	1.70 (0.85 - 3.39)	0.55 (0.25 - 1.21)			
Africa	Southern and Eastern Africa	29	1098	2.64 (1.84 - 3.80)	0.86 (0.51 - 1.45)			
	Middle, Western and Northern Africa	26	532	4.89 (3.33 - 7.18)	1.58 (0.92 - 2.71)			
Asia	South-Eastern and Eastern Asia	69	2625	2.63 (2.08 - 3.33)	0.85 (0.55 - 1.33)			
	South-Central and Western Asia	18	871	2.07 (1.30 - 3.28)	0.67 (0.37 - 1.22)			
Several regions	Several regions	7	199	3.52 (1.68 - 7.38)	1.14 (0.50 - 2.62)			

**Figure 2:**
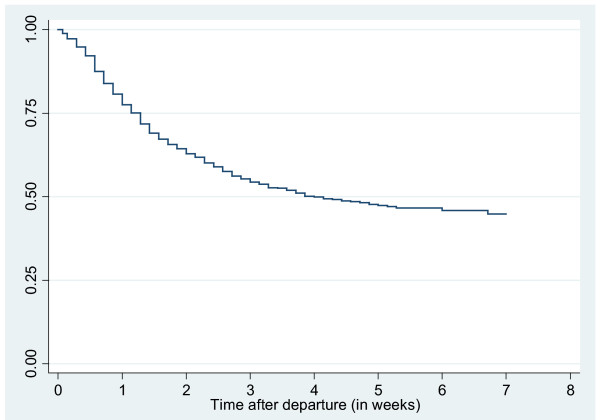
**Kaplan-Meier graph of diarrhoea-free survival among 1202 short-term travellers from the Netherlands to developing countries, 2006-7**. Time is indicated in weeks and data censored at 7 weeks.

Of first episodes, 228 out of 597 (38%) started in the first travel week, and most of the subsequent episodes (113 out of 184, 61%) started after the third week of travel. Time until the first episode of TD is shown in Figure 2.

The incidence rate for subsequent episodes was highest in Middle, Western, and Northern Africa (4.89, 95% CI 3.33 - 7.18) (Table 3). The only independent risk factor for a subsequent episode of TD was female sex (IRR 1.50, 95% CI 1.09 - 2.04) (Table 3).

During first episodes of TD, fever was reported by 85 of 597 subjects (14%), vomiting by 75 (13%), nausea by 5 (6%), and abdominal cramps by 31 (5%). During subsequent episodes of TD in 184 cases, fever was reported by 20 (11%), vomiting by 11 (6%), nausea by 18 (10%), and abdominal cramps by 12 (7%). No accompanying symptoms were reported in 428 (72%) of first episodes and in 137 (75%) of subsequent episodes. Of all 781 TD episodes, 29 (4%) were considered severe, and the attack rates of severe TD were not different for first and subsequent episodes (p = 0.940; AR_first episodes _4% (22/597), AR_subsequent episodes _4% (7/184)). None of the variables we examined were associated with severe episodes of TD.

### Treatment

Of all 781 subjects with episodes of diarrhoea, 464 (59%) took no treatment, 231 (30%) used an antimotility agent such as loperamide, 116 (15%) oral rehydration solution (ORS), and 36 (5%) antibiotic treatment: 33 out of 752 (5%) for mild TD and 3 of 29 (10%) for severe TD (Table 4). Of the 36 subjects that used antibiotic treatment, 18 also used an antimotility agent, for mild diarrhoea. Of all 781 travellers who had TD, 25 (3%) consulted a physician, including 2 with severe disease. For 17 out of the 25 who sought medical treatment, local physicians prescribed antibiotics. The other 19 subjects (53%) who used antibiotics bought them over the counter.

**Table 4:** Treatment for travellers diarrhoea in a prospective cohort of 1202 travellers from the Netherlands to developing countries, October 2006 - October 2007

Treatment	Total episodes		1st episode		2nd and subsequent episodes	
			**mild**	**severe**	**mild**	**severe**
Total	781		575 (96%)	22 (4%)	177 (96%)	7 (4%)
						
No treatment	464	59%	327 (57%)	11 (44%)	123 (70%)	3 (43%)
Antimotility agents	231	30%	178 (31%)	7 (32%)	46 (26%)	0
ORS	116	15%	92 (16%)	6 (27%)	16 (9%)	2 (29%)
Antibiotic	36	5%	30 (5%)	1 (5%)	3 (2%)	2 (29%)
Alternative antidiarrhoeal drugs	22	3%	18 (3%)	1 (5%)	3 (2%)	0
Visited physician for diarrhoea	25	3%	18 (3%)	1 (5%)	5 (3%)	1 (14%)

## Discussion

Despite the pre-travel advice on personal hygiene measures to prevent travellers' diarrhoea, it still occurred in half of all travellers. Compliance with these measures and, therefore, the effectiveness of the recommendations has been found to be poor [6,20]. The high risk of TD found in our study is comparable with earlier prospective studies, which have found attack rates to vary between 25% and 57% [4,9,11,21,22].

In our study, the IR and AR were highest in South-Central (the Indian subcontinent) and Western Asia, followed by Middle, Western and Northern Africa and Central America and the Caribbean. Most other studies have also found the Indian subcontinent to be a high-risk area, followed by African regions [2-4,9,11,23,24]. Findings of AR and IR in different regions within Latin America differ between studies [3,4,9,11,23,24]. However, comparing risk patterns between destinations in various studies is difficult, because of differing study methods and definitions for travellers' diarrhoea and for regions. The regional variations in AR and IR may be due to differences in circulating pathogens [8] and in hygienic standards between countries [25].

Of all 781 episodes of TD in our study, only 29 (4%) were considered severe, as defined by blood and/or mucous content. Possibly these episodes were caused by one of the invasive pathogens, like *Campylobacter jejuni*, shigella or salmonella species. As in other studies, most TD episodes were mild. Cobelens et al. [3] found fecal blood loss and concomitant abdominal and systemic symptoms in subsequent episodes more often than in first episodes and subsequent episodes lasted longer than first. However, we found no difference in severity of TD between first and subsequent episodes and subsequent episodes had a significant shorter median duration than first episodes. Because it is unlikely that exposure to pathogens decreases, this could possibly mean that travellers acquire immunity to some pathogens, similar to people born in non-Western countries.

Baaten et al. [25] found that the incidence of other feco-orally transmitted infections like hepatitis A, shigellosis, and typhoid fever among travellers declined in the past 10 years, which seemed related to an increase in hygienic standards in the destination countries. On that basis, we would expect the incidence of TD to have declined over the last decades. Indeed, the overall incidence rate of TD in our study of 2.49 per 100 travel days (95% CI 2.30 - 2.70) was significantly lower compared to the IR of 3.14 per 100 travel days (95% CI 2.86 - 3.43) observed 10 years ago among travellers in Amsterdam [3]. However, these two studies were different in some respects. First, because the travellers in our study had different travel patterns, we defined somewhat larger regions. Second, we used the WHO definition of TD, whereas Cobelens defined TD as any episode of 3 or more unformed stools daily or any number of such bowel movements accompanied by vomiting, abdominal cramps, or subjective fever, with an onset between the beginning and end of the journey. Finally, our study was prospective, with travellers asked to record symptoms daily in a diary, whereas Cobelens' study was retrospective, and thus may be subject to recall bias. Although we cannot conclude that the IR of TD has actually decreased, the finding of a lower IR with a broader definition could indicate that it is indeed declining.

Independent risk factors for first episodes of TD in our study were female sex, a Western country of birth, tourism as the purpose of travel, and some travel destinations. Also, female sex was an independent risk factor for subsequent episodes of TD. Sex differences in travel-associated disease and diarrhoea have been reported previously [26], but it is unclear if this difference can be explained by different travel behavior or by women being more susceptible to diarrhoea or more likely to report diarrhoea. Birth in a non-Western country may confer immunity to TD because of increased exposure to infections endemic in those countries [2,27]. Several studies have shown an increased risk of TD in younger age groups [5,6,9]; we also found an association between younger age and the risk of TD, although not significant.

To our knowledge, our study is the first to calculate IR for subsequent episodes. Incidence rates did not differ between the development of a first episode of TD and a later episode, and neither did we find differences in severity between the first and subsequent episodes. Apparently, having had an episode of diarrhoea is not protective for a subsequent episode. Although antibiotic stand-by treatment was not prescribed before travel, only 5% of travellers with TD resorted to antibiotics purchased locally. Prescribing them to all would leave millions of courses of unused antibiotics in circulation. Aside from the unnecessary cost to travellers, public health services, and/or insurers, improper use can contribute to increased antimicrobial resistance.

Some people might argue that the use of antibiotics would result in a shorter duration of diarrhoea, as was found in a recent Cochrane meta-analysis [28]. The end-point of this placebo controlled analysis was duration of diarrhoea less than 72 hours, and severity. As our study showed an overall mild course of TD, and a short median duration of TD of 48 hours, it remains questionable whether antibiotics would have benefited our group of travellers, especially since the occurrence of side effects of antibiotics as shown by the same analysis.

The strength of our study is that it is prospective, which is the best approach for estimating attack rates and incidence rates of travellers' diarrhoea. Another strength is the daily diary entries, which minimizes recall bias. Our study also had some possible limitations. We used the WHO definition of TD (any number of more frequent passage of loose or liquid stools per day than is normal for the individual), and asked for accompanying symptoms to define the severity of diarrhoea. We did not ask participants to record the frequency of stools per 24 hr nor the degree of disability that travellers experienced, which would have possibly allowed us to make more detailed analyses and compare our results with more studies. On the other hand, asking holiday makers to fill out too many details could lead to less compliance with our study.

## Conclusions

Diarrhoea is still a very frequently contracted infectious disease by travellers, but the overall course of TD appears to be mild, not requiring treatment. Prescriptions for stand-by antibiotics should be limited to travellers at high risk, such as those who are immunocompromised or are visiting very remote areas.

## Competing interests

The authors declare that they have no competing interests.

## Authors' contributions

SB analysed the data and wrote the article. GS designed the study and contributed to the article. MS analysed the data and contributed to the article. JK collected and analysed data. AvdH designed the study, contributed to the article and was guarantor.

All authors read and approved the final manuscript.

## Pre-publication history

The pre-publication history for this paper can be accessed here:

http://www.biomedcentral.com/1471-2334/11/295/prepub
